# Calreticulin is required for development of the cumulus oocyte complex and female fertility

**DOI:** 10.1038/srep14254

**Published:** 2015-09-21

**Authors:** Keizo Tokuhiro, Yuhkoh Satouh, Kaori Nozawa, Ayako Isotani, Yoshitaka Fujihara, Yumiko Hirashima, Hiroyuki Matsumura, Kazuhiro Takumi, Takashi Miyano, Masaru Okabe, Adam M. Benham, Masahito Ikawa

**Affiliations:** 1 Research Institute for Microbial Diseases, Osaka University, Suita, Osaka, Japan; 2 Graduate School of medicine, Osaka University, Suita, Osaka, Japan; 3 Immunology Frontier Research Center, Osaka University, Suita, Osaka, Japan; 4 Graduate School of Pharmaceutical Sciences, Osaka University, Suita, Osaka, Japan; 5 Graduate School of Agricultural Science, Kobe University, Kobe, Hyogo, Japan; 6 School of Biological and Biomedical Sciences, Durham University, Durham, United Kingdom

## Abstract

Calnexin (CANX) and calreticulin (CALR) chaperones mediate nascent glycoprotein folding in the endoplasmic reticulum. Here we report that these chaperones have distinct roles in male and female fertility. *Canx* null mice are growth retarded but fertile. *Calr* null mice die during embryonic development, rendering indeterminate any effect on reproduction. Therefore, we conditionally ablated *Calr* in male and female germ cells using *Stra8* (mcKO) and *Zp3* (fcKO) promoter-driven Cre recombinase, respectively. *Calr* mcKO male mice were fertile, but fcKO female mice were sterile despite normal mating behavior. Strikingly, we found that *Calr* fcKO female mice had impaired folliculogenesis and decreased ovulatory rates due to defective proliferation of cuboidal granulosa cells. Oocyte-derived, TGF-beta family proteins play a major role in follicular development and molecular analysis revealed that the normal processing of GDF9 and BMP15 was defective in *Calr* fcKO oocytes. These findings highlight the importance of CALR in female reproduction and demonstrate that compromised CALR function leads to ovarian insufficiency and female infertility.

Proper folding in the endoplasmic reticulum (ER) is a prerequisite for correct localization and function of most secreted and transmembrane proteins^1^. Failures in protein folding and quality control compromise cellular functions and cause disease including amyloidosis, cystic fibrosis, and diabetes^2^,^3^. Failure in ER quality control also results in male infertility^4^,^5^,^6^. In the ER, soluble calreticulin (CALR) and membrane bound calnexin (CANX) were originally discovered as homologous calcium binding proteins and later shown to be lectin like chaperones that chiefly mediate nascent glycoprotein folding^7^,^8^,^9^. Despite their extensive homology, CALR and CANX have contrasting functions. For example, proteins in *Calr*^−/−^ cells have accelerated folding with an accumulation of misfolded proteins whereas folding is significantly impaired in *Canx*^−/−^ cells^10^. Differences in CALR and CANX function are further reflected in the distinct phenotypes of knock-out mice. *Calr*^−/−^ mice are embryonic lethal due to defective heart development whereas *Canx*^−/−^ mice are viable but growth retarded with neurological deficits^11^,^12^,^13^. However, the client specificity of calnexin/calreticulin *in vivo* has not been fully established.

We have previously demonstrated that calmegin (CLGN) and calsperin (CALR3) are testis specific homologues of CANX and CALR, respectively, and are expressed in the ER of spermatogenic cells^4^,^5^,^14^. CLGN mediates the heterodimerization of ADAM1A/ADAM2 that is required for the maturation of ADAM3, a sperm membrane protein. In contrast to this indirect activity, CALR3 binds directly to ADAM3 and regulates its maturation. Although a pseudogene in humans^15^, mouse ADAM3 is essential for sperm migration from the uterus into the oviduct and *Adam3* null male mice are sterile^16^,^17^. Both *Clgn* and *Calr3* null mice lack ADAM3 on their sperm surface and null males are infertile. However, other membrane and secretory proteins are normally present in these mutant spermatozoa, indicating the importance of CLGN/CALR3 rather than CANX/CALR, for the maturation of ADAM3 related proteins^4^,^5^. The restricted client specificity of CLGN and CALR3 is further highlighted by the fact that the N-glycosylated sperm membrane fusion protein IZUMO1 is functionally presented on *Clgn*/*Calr3* mutant spermatozoa^18^.

CLGN and CALR3 are not expressed in the ovary and thus female germ cells rely on the CANX/CALR system for quality control of nascent proteins in the ER. During oogenesis, oocytes secrete many factors that regulate the growth and differentiation of granulosa cells, including GDF9 and BMP15. These two proteins are structurally complex with extensive disulfide-bonds and N-linked glycans. Mutations that alter their ability to fold have been implicated as causes of premature ovarian failure^19^,^20^,^21^,^22^. *Gdf9*^−/−^ and *Bmp15*^−/−^ females are sterile or subfertile, respectively^23^,^24^ and a recent paper has reported the physiological importance of the GDF9:BMP15 heterodimer^25^.

In the present study, we have established mouse lines in which either *Canx* or *Calr* has been ablated to investigate their roles in male and female germ cell development and function. Although growth retardation was observed, both male and female *Canx* knockout mice were fertile. For CALR, we generated male and female germ cell specific knockout mice to circumvent the embryonic lethality. Whereas CALR was dispensable for spermatogenesis and sperm fertilizing ability, it was required in the oocyte for the maturation of the TGF-beta family proteins, GDF9 and BMP15, as well as the subsequent development of the cumulus oocyte complex (COC). Our results highlight the importance of CALR for female reproduction and suggest that compromised CALR function can lead to ovarian insufficiency and female sterility.

## Results

### Generation of conditional knockout (KO) mice for Canx and Calr genes

**Figure 1:**
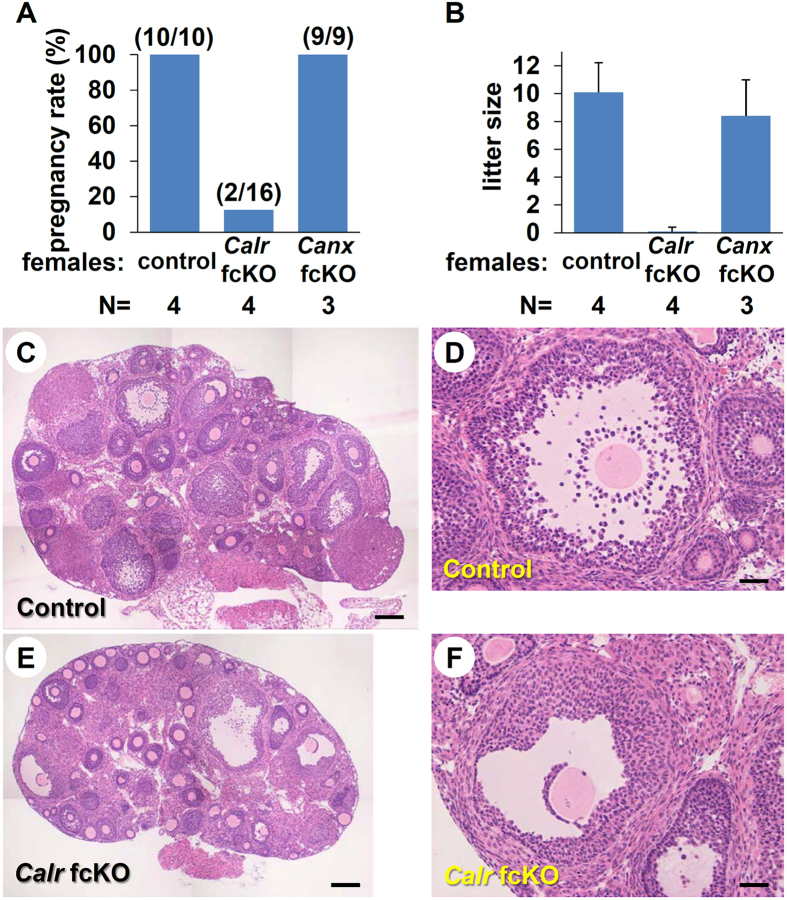
Abnormal ovarian follicular development in *Calr* fcKO mice. (**A**) Pregnancy rate (pregnancy/vaginal plug formation) obtained by mating control (*Calr*^fl/+^; *Zp3-cre*), *Calr* fcKO (*Calr*^fl/−^; *Zp3-cre*) and *Canx* fcKO (*Canx*^fl/−^; *Zp3-cre*) females with B6D2F1 wild-type male mice. The total number of plugs observed is indicated in parentheses. (**B**) Average litter sizes obtained by mating control, *Calr* fcKO and *Canx* fcKO females with B6D2F1 wild-type male mice. (**C**–**F**) Histological analysis of ovarian sections. Preovulatory follicles from *Calr* fcKO mice demonstrate immature follicles just under the surface of the ovary. The cumulus mass surrounding oocytes is composed of fewer cells than control. Scale bar = 300 μm (**C**,**E**) and 50 μm (**D**,**F**). Error bars represent standard deviation (**B**).

Mice with genetically disrupted *Canx* and *Calr* genes are growth retarded or die during embryonic development, respectively^11^,^12^,^13^. Thus, we generated male (mcKO) and female (fcKO) germ cell-specific conditional knockout mice to investigate the role of these genes in fertility. We first generated mice carrying floxed alleles (fl/+) using gene targeting vectors that floxed exons 3 and 4 for *Canx* and exons 4–7 for *Calr* (Supplementary Fig. S1A and Supplementary Fig. S2A, related to Fig. 1). The correct gene targeting event in embryonic stem cells and subsequent germ-line transmission was confirmed by PCR analysis (Supplementary Fig. S1B and Supplementary Fig. S2B). To remove the floxed exons in male and female germ cells, we used transgenic mouse lines expressing Cre recombinase under the *Stra8* and *Zp3* promoters, respectively^26^,^27^. Homozygous knockout (−/−) mice were generated by crossing heterozygous mutant (+/−) mice.

### Fertility in Canx knockout mice

When we crossed *Canx*^+/−^ females with *Canx*^+/−^ males, *Canx*^−/−^ mice were born in expected Mendelian ratios (+/+:+/−:−/−=14:27:16, n = 8 litters from 3 breeding pairs). However, 50% of the homozygous null pups died within 48 hours and very few survived to three months, as reported previously^12^. Similar postnatal lethality was not observed in *Canx* gene trapped mice despite the complete absence of CANX protein^13^. Paradoxically, truncated CANX protein was present in one of the two lines in the earlier report with the more severe phenotype. Using antibodies against the N- and C- terminal residues, CANX protein was not detected in our −/− mice (Supplementary Fig. S1D). Therefore the variance in postnatal viability reported earlier may reflect the combined effect of CANX deficiency and other factors, including genetic background and animal husbandry.

To examine *Canx*^−/−^ mouse fertility, adult (8 week-old) *Canx*^−/−^ females and males were mated with wild-type (+/+) males and females, respectively. Although the testis size was smaller in *Canx*^−/−^ male mice, the ratio of testis weight to body weight was comparable to that of wild-type (0.37 ± 0.03% and 0.39 ± 0.06%, respectively) and normal spermatogenesis was observed (Supplementary Fig. S3, related to Fig. 1). *Canx*^−/−^ males were fertile and average litter sizes were 7.1 ± 2.2 and 7.5 ± 0.8 (avg. ± s.d. pups, n = 6 litters from 3 males) in normal and null mice respectively.

*Canx*^−/−^ females were also fertile, but litter sizes were smaller (3.3 ± 1.5 pups, n = 4 litters from 4 females) possibly due to their smaller body size. We produced oocyte specific *Canx* fcKO mice by introducing the *ZP3-cre* transgene (*Canx*^fl/−^; *Zp3-cre*). Mice were genotyped by PCR analysis and the lack of CANX protein was confirmed by immunoblot (Supplementary Fig. S1C, related to Fig. 1). *Canx* fcKO female mice had normal fertility when mated with wild type males (8.4 ± 2.6 pups, n = 9 litters from 3 females) (Fig. 1A,B). All 76 pups carried the knockout allele, which confirmed successful excision of the floxed exons by the *Zp3* promoter driven Cre recombinase. Therefore, we conclude that CANX is not required for either male or female reproduction.

### Female infertility in Calr conditional knockout mice

It was reported that the *Stra8* promoter driven Cre recombinase was expressed at the postnatal day 3 in early-stage spermatogonia and the recombination efficiency was >95%^26^. In the present study, with our *Stra8-cre* transgenic line, *Calr* disruption was confirmed in most (84.2%, 219/260) of the testicular germ cells as determined by immunostaining (Supplementary Fig. S4A and B, related to Fig. 1). When mated with normal female mice, *Calr* mcKO males had comparable fertility (10.1 ± 1.5 pups, n = 26 litters from 6 males) with normal male mice (10.6 ± 1.3 pups, n = 8 litters from 3 males). Whereas some pups inherited the floxed allele, the majority (82.1%, 215/262) of the offspring inherited the knockout allele, which was consistent with the aforementioned observations in testicular germ cells.

We next used *ZP3-cre* transgenic lines to disrupt the *Calr* gene during oogenesis (Supplementary Fig. S2C and Supplementary Fig. S4C, related to Fig. 1). Whereas control female mice (*Calr*^fl/+^; *Zp3-cre*) had normal fertility, *Calr* fcKO female mice had a profound decrease in fecundity (Fig. 1A,B). Only 2 litters with 1 pup each were obtained from 16 copulations with 4 *Calr* fcKO females, whereas 10 litters from 10 copulations were obtained with 4 control females. The average litter sizes were 0.1 ± 0.3 and 8.4 ± 2.6 pups, respectively. When we superovulated *Calr* fcKO females with gonadotropins, successful copulation was observed. Thus, the female infertility was not caused by disrupted mating behavior. However, ovulation was severely impaired in the *Calr* fcKO female mice (Table 1).

To elucidate the cause of infertility in the *Calr* fcKO females, we superovulated 3 (Fig. 1C–F) or 12 (Supplementary Fig. S5C–H) week-old female mice and prepared ovarian sections 2 hours before the anticipated time of ovulation. In *Calr* fcKO mice, the ovary was smaller, possibly due to defective folliculogenesis (Table 2). A few preovulatory follicles appeared, but most of the follicles at the surface of the ovary were immature. Follicular development was arrested at the early antral stage, the number of cumulus cells that surrounded an oocyte after germinal vesicle breakdown in cross sections was reduced (33.1 ± 13.6 in fcKO and 141.6 ± 30.5 in control) and cumulus expansion was impaired (Fig. 1C–F and Supplementary Fig. S5A–H). Corpora lutea were rarely observed in *Calr* fcKO ovaries. Because antral follicles were present, we assayed *in vitro* maturation and *in vitro* fertilization. Comparable numbers of oocytes were collected from control and *Calr* fcKO mice 46–48 hours after PMSG injection. Both control and mutant oocytes underwent germinal vesicle breakdown (GVBD) and matured to the metaphase II (MII) stage *in vitro*. The oocytes collected from the *Calr* fcKO had slightly larger diameters than those collected from control mice (77.6 ± 0.3 μm in fcKO and 73.5 ± 0.4 μm in control, *P* < 0.01), as reported in *Gdf9* KO mice^23^. Although the efficiency was lower than in controls, these MII eggs could be fertilized and developed to term after transfer into pseudopregnant females (Table 3).

### CALR mediated quality control of GDF9 and BMP15

**Figure 2:**
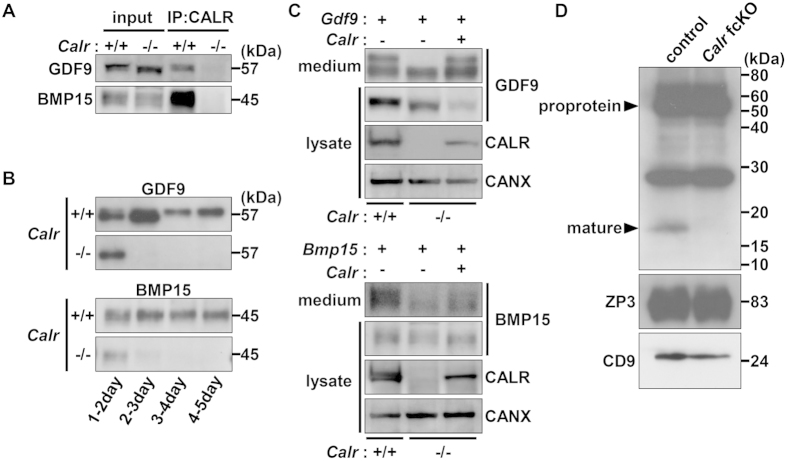
CALR is essential for extracellular secretion of GDF9 and BMP15. (**A**) *Calr*^+/+^ or *Calr*^−/−^ MEFs expressing Flag/His tagged GDF9 or BMP15 were immunoprecipitated (IP) with anti-CALR antibody from the cell lysates (50 μg) and the immunoprecipitates were probed with anti-His antibody on immunoblots. (**B**) Secreted GDF9 and BMP15 from *Calr*^+/+^ or *Calr*^−/−^ MEFs were examined every 24 hours after transfection. *Calr*^−/−^ MEFs stopped secreting these proteins after 1–2 days. (**C**) Secreted GDF9 and BMP15 were both observed as a single band in *Calr*^−/−^ MEFs. The upper band was recovered by co-transfection with a plasmid expressing *Calr.* (**D**) The 57 kDa GDF9 proprotein was present, but not the 17.5 kDa mature GDF9 in CALR deficient oocytes. Other glycoproteins, CD9 and ZP3, were normally expressed. 300 oocytes (GDF9) or 20 oocytes (ZP3 and CD9) were loaded per lane.

*Calr* fcKO had impaired ovulation with defects in cumulus expansion. GDF9 and BMP15 are two growth factors that are secreted from oocytes and stimulate cumulus cell expansion^23^,^28^. To examine the effect of CALR disruption on the production of these proteins, primary mouse embryonic fibroblast (MEF) cells were prepared from *Calr*^+/+^ and *Calr*^−/−^ mice and transfected with mouse *Gdf9* or *Bmp15* expression vectors. Using co-immunoprecipitation, we confirmed that CALR associates with GDF9 and BMP15 in wild-type cells (Fig. 2A). In *Calr*^+/+^ MEFs, GDF9 and BMP15 were secreted into the extracellular fluid for 4–5 days, whereas in *Calr*^−/−^ MEFs, secretion stopped within 1–2 days (Fig. 2B). When carefully observed, GDF9 proproteins appeared as a doublet on SDS-PAGE^29^, but the upper band was not present in *Calr*^−/−^ cell supernatants (Fig. 2C). Recovery of the doublet was observed after co-transfection of a *Calr* expression vector. Similar results were observed for BMP15. To examine whether GDF9 is expressed and/or secreted in *Calr* fcKO oocytes, we collected 300 oocytes and examined their lysates by immunoblot (Fig. 2D). Whereas comparable amounts of GDF9 proproteins (57 kDa) were observed in control and fcKO oocytes, mature type GDF9 (17 kDa) was detected in control, but not in CALR deficient oocytes. There were comparable amounts of other fertilization related N-glycoproteins, including ZP3 and CD9.

### Complementation of defective GOC (granulosa-oocyte complex) development by recombinant GDF9 and BMP15

**Figure 3:**
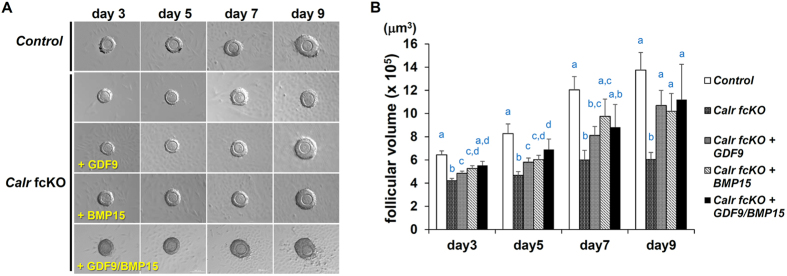
Recombinant GDF9 and BMP15 partially restored follicular development of *Calr* cKO. (**A**) Control and *Calr* fcKO follicles were collected from 12 day-old mice and cultured *in vitro* for 9 days. In *Calr* fcKO mice, the addition of GDF9 or BMP15 partially promoted granulosa cell proliferation and increased the follicular volume of *Calr* fcKO. (**B**) The average follicular volumes from three independent experiments were determined. Error bars represent standard error of the mean. Columns with the same letter are not significantly different (P > 0.05). (n = 43, 39, 43, 38 and 12 for Control (*Calr*^fl/+^; *Zp3-cre*), *Calr* fcKO, *Calr* fcKO+GDF9, *Calr* fcKO+BMP15 and *Calr* cKO+GDF9/BMP15, respectively).

External addition of recombinant BMP15 or GDF9 improves cumulus cell proliferation, differentiation, and steroidogenesis in *Bmp15/Gdf9* null mice^30^,^31^. In the present study, we examined whether GDF9 and BMP15 supplements could restore the defective GOC development in *Calr* fcKO mice using an *in vitro* follicle culture system. Without supplementation, granulosa cells proliferated and the size of the follicles gradually increased when CALR was present. In *Calr* fcKO GOC, the follicle size did not increase compared to controls (Fig. 3). When the GOC growth medium was supplemented with either recombinant GDF9 or BMP15, both factors significantly enhanced *Calr* fcKO GOC growth. Conditioned medium from cells expressing both GDF9 and BMP15 did not show a synergistic effect.

## Discussion

Although CANX is not essential for mouse development *in vivo*^12^,^13^, it has been difficult to investigate its role in the reproductive system since few *Canx* KO mice survived to adulthood and they were smaller than their littermates. In the present study, we showed that *Canx* KO males were able to copulate and successfully impregnate females. *Canx* KO females showed reduced litter size, but oocyte specific disrupted cKO females were fully fertile. Therefore we conclude that CANX is dispensable for both male and female reproductive systems.

We next examined the role of CALR, the soluble homologue of CANX, in the reproductive system. *Calr* KO mice are embryonic lethal but ectopic expression of calcineurin in the heart enabled *Calr* KO mice to survive until adulthood^32^. However, the surviving mice exhibited growth retardation, hampering any study of their reproduction. Here, we generated male germ cell specific cKO mice and showed that CALR is dispensable for spermatogenesis and sperm fertilizing ability. Although CANX and CALR have contrasting functions in other tissues^12^,^32^, their major roles may be redundant and complementary to each other, at least in developing male germ cells. In contrast, the homologues of CANX and CALR, CLGN and CALR3, respectively, have different substrate specificity and both, albeit by different mechanisms, are required for fertilization by controlling ADAM3 presentation on the sperm surface^4^,^5^. Thus, the present study reinforces the uniqueness and importance of the CLGN/CALR3 ER chaperone system in the male reproductive system.

In the female reproductive system, we have shown that CALR has a novel and indispensable role in COC development and female fertility. Since GDF9 and BMP15 are secreted proteins, soluble CALR might be more accessible to these molecules than membrane-tethered CANX. However it is also reported that soluble CANX and CALR have different substrate specificity^33^ and an in-depth analysis of the molecular interactions of these chaperones with these two growth factors requires future investigation. Our data indicated CALR plays an important role in regulating the folding of GDF9 and BMP15 in the ER. The folding defects could cause various effects, such as protein instability, secretion defects, abnormal cleavage or aberrant post-translational modifications of GDF9 and BMP15 at later stages of the secretory pathway.

In the present study we could not detect mature GDF9 and BMP15 proteins in MEF cells. The defect in the processing machinery may cause different behaviors in unfolded GDF9 and BMP15 in MEF and oocytes. It has been reported that the mature recombinant mouse BMP15 is not processed in HEK 293T cells and CHO cells^34^. Further, the post-translational processing of BMP15 is precisely regulated during meiosis and the mature BMP15 can be detected 9 hours after the hCG injection in mice^28^. These studies imply that oocytes may have a unique post-translational processing mechanism for GDF9 and BMP15.

The follicular development in the *Gdf9* KO is arrested at the primary stage and the *Bmp15* KO showed normal folliculogenesis despite decreased ovulation and rates of fertilization^23^,^24^. However, follicular development in *Calr* fcKO mice arrested at the early antral stage. In *Calr* fcKO mice, GDF9 proprotein is expressed in oocytes, but not cleaved. In rat, macaque and human, GDF9 proprotein or BMP15 is detected in the follicular fluid^35^,^36^. In addition, mutation in the protease cleavage site of GDF9 causes female infertility in ewes^37^. The hypoplastic ovaries of homozygous mutated ewe lambs contain large numbers of primordial follicles and developing follicles up to the early antral stage. These data suggest that the proprotein of GDF9 accounts for the different phenotypes in *Gdf9* KO and *Calr* fcKO mice.

The mechanism of COC development has been well investigated and many key factors have been identified in both oocyte and surrounding granulosa cells^38^,^39^. Among these factors, GDF9 and BMP15 are secreted from developing oocytes and stimulate granulosa cell proliferation and expansion^24^,^31^,^40^. GDF9 and BMP15 have a unique disulfide bond arrangement among TGF-beta family proteins^41^,^42^,^43^, and showed different mobility in SDS electrophoresis under reducing or non-reducing conditions^31^,^44^, indicating the importance of disulphide bond formation during their proper folding, targeting, and function. Here we showed that CALR interacts with GDF9 as well as BMP15 and the lack of CALR compromised their secretion. This is reminiscent of the misfolding and disappearance of ADAM3 in *Calr3* knockout male mice. In testis, CALR3 recruits PDILT (protein disulfide isomerase like in testis) and assists in the quality control of ADAM3^6^. From studies of other nascent glycoproteins folding in somatic cells^45^,^46^,^47^, CALR may cooperate with PDIA3 (ERP57) to regulate the quality control of GDF9/BMP15.

GDF9 and BMP15 play important roles in energy metabolism and/or cholesterol biosynthesis in granulosa cells^25^,^48^,^49^. Because TGF-beta/Smad3 signaling regulates glucose and energy homeostasis, and *Smad3* KO mice exhibit improved glucose tolerance and enhanced glucose-stimulated insulin secretion from pancreatic islet beta cells^50^,^51^, it will be interesting to investigate the role of CALR in the secretion of TGF-beta family proteins in other tissues. That might also explain why *Calr* KO mice, transgenically rescued from embryonic lethality, still die mainly due to metabolic failure^32^. Of interest, it was also reported that TGF-beta stimulates cells and induces extracellular matrix secretion that depends on CALR mediated Ca^2+^ signaling^52^. Thus the ability of CALR to regulate calcium availability might also be important downstream of the TGF-beta signaling pathway. In the present study, we could not fully recover GOC development with recombinant GDF9/BMP15 (Fig. 3), implying that there are other CALR dependent factors required for complete oocyte development.

In conclusion, we document that CALR plays an indispensable role in the COC development by controlling the maturation of the TGF-beta proteins GDF9 and BMP15. Because GDF9 and BMP15 are known to be involved in premature ovarian failure and polycystic ovarian syndrome^22^,^53^,^54^,^55^, targeting CALR and the ER quality control system in follicular development should be considered as a novel avenue for female infertility treatment.

## Methods

### Animal experimentation

All animal experiments were carried out in accordance with the protocols approved by the Animal Care and Use Committee of the Research Institute for Microbial Diseases, Osaka University. The *Stra8*-cre mouse line was established in our laboratory by injecting a transgene that was constructed by replacing CAG promoter in the pCX-cre vector with the *Stra8* promoter^56^. The *Zp3-cre* mouse line was obtained from the Jackson laboratory^27^.

### Generation of KO and cKO mice

Targeting vectors were constructed by placing *Canx* genomic fragments (1.7 kbp, 2.0 kbp and 4.0 kbp) and *Calr* genomic fragments (2.0 kbp, 1.4 kbp and 3.5 kbp) into pNT1.1^57^. D3 embryonic stem cells were electroporated with the NotI-linearized targeting vector. After G418/GANC selection, drug resistant clones (3/96 for *Canx* and 5/672 for *Calr*) with homologous recombinants (tm1a) were identified by PCR analysis. Three targeted clones were injected into C57BL/6 blastocysts, resulting in the birth of coat-color chimeric male mice. The neomycin-resistant cassette was removed by crossing with transgenic mice expressing FLPe under the CAG promoter and this allowed the generation of mice with the conditional floxed (fl or tm1b) allele (*Canx*^fl/+^ or *Calr*^fl/+^). These mice were crossed with transgenic mice expressing Cre under the CAG promoter to generate *Canx*^+/−^ or *Calr*^+/−^ mice (- or tm1c)*. Canx*^−/−^ or *Calr*^−/−^ mice were generated by mating with each heterozygous mutated mouse that did not contain CAG-FLPe and CAG-Cre. To generate cKO mice, *Canx*^fl/+^ or *Calr*^fl/+^ mice were crossed with transgenic mice expressing Cre under the control of *Stra8* or *Zp3* promoters. For *Canx* fcKO, *Canx*^fl/+^; *Zp3-cre* mice were crossed with *Canx*^fl/−^ to produce mice with the following genotype: *Canx*^fl/+^; *Zp3-cre* (for control) and *Canx*^fl/−^; *Zp3-cre* (for fcKO). *Calr* cKO were generated through the same breeding strategy as *Canx* cKO using *Stra8-cre* or *Zp3-cre* transgenic mice. Pr-FlpeF; 5′- ccacctaaggtcctggttcgtcagtttgtg -3′ and pr-FlpeR; 5- atacaagtggatcgatcctaccccttgcgc -3′ primers were used for the *Flpe* transgene in addition to the primers indicated in Figure S1 and Figure S2.

### Histology of the ovary

Female mice were injected with pregnant mare serum gonadotropin (PMSG) and human chorionic gonadotropin (hCG) at 48 hour intervals. Ovaries were collected at 10 hours after hCG injection and fixed with 4% paraformaldehyde/PBS overnight. Then the ovaries were embedded into glycol methacrylate (Technobit 8100: Heraeus Kulzer, Germany) after treatment with a graded ethanol series. Plastic thin sections (5 μm) were stained with hematoxylin and eosin. For Periodic acid Schiff (PAS) staining, sections were rehydrated, and treated with 2% metaperiodic acid for 15 minutes, followed by treatment with Schiff’s reagent (Wako, Japan) for 20 minutes. The sections were stained with hematoxylin prior to imaging.

### Immunohistochemistry

Ovaries were collected from adult mice and fixed in 4% (wt/vol) paraformaldehyde/PBS overnight at 4 °C, cryopreserved in graded 10–30% (wt/vol) sucrose, and embedded in TissueTek OCT compound (Sakura Finetechnical). Frozen sections (5 μm) were mounted on APS (aminosilane)-coated glass slides. After washing with PBS, slides were blocked with 10% (vol/vol) newborn calf serum (NBCS)/PBS for 1 hour and incubated with anti CALR antibody in 10% (vol/vol) NBCS/PBS at 4 °C overnight. After washing with PBS, the slides were incubated with secondary antibodies with Alexa Fluor 488 (Invitrogen) in 10% (vol/vol) NBCS/PBS for 2 hours. After washing with PBS, the slides were imaged with an Olympus IX-70 fluorescence microscope.

### *In vitro* maturation and fertilization

For *in vitro* maturation^58^, immature GV (germinal vesicle) oocytes were collected from ovaries 46 hours after injection with PMSG. Antral follicles were punctured with 26G needles in FHM^59^ with 100 μM dibutyryl-cyclic AMP (Sigma). After dissociating the cumulus cells by pipetting, oocytes were washed 3 times and cultured in Minimum Essential Medium Alpha (GIBCO) with 3 mg BSA (A3311, Sigma). After 14 hours, partial zona dissection of MII oocytes was performed using a piezo-micromanipulator with a glass capillary needle (diameter of 5–10 μm)^60^ and incubated in TYH medium^61^ with 2 × 10^5^/ml B6D2F1 capacitated sperm. Fertilization was determined by formation of two-cell embryos, which were then transferred into the oviduct of pseudopregnant mice.

### Immunoblot and immunoprecipitation

Oocytes were collected from ovaries 46 hours after PMSG injection. Cumulus or granulosa cells were dissociated using glass pipettes and collected oocytes were boiled in SDS-PAGE sample buffer. Proteins were separated by SDS-PAGE and run under reducing conditions. Immunoprecipitation was performed as described^5^. Antibodies used included rabbit antisera against CAXN and CALR^5^, rabbit antibody against the N-terminus of CAXN (sc-11397, Santa Cruz Biotechnology), penta-His mouse monoclonal antibody (Qiagen), horseradish peroxidase conjugated goat anti-rabbit IgG and goat anti-mouse IgG antibodies (Jackson Immuno Research Laboratories); and a GDF9 monoclonal antibody^62^, kindly provided by Dr. Martin M. Matzuk.

### Preparation of *Calr* deficient mouse embryonic fibroblasts (MEF)

MEFs were isolated from 13.5–14.5 day-old embryos. The head and all internal organs were removed from the embryo. The remaining tissue was minced with scissors and incubated in 0.25% trypsin containing penicillin (100 U/ml, Nacalai) and streptomycin (100 μg/ml, Nacalai) for 10–20 min. The cells were pipetted and plated onto a 10 cm tissue culture dish in culture medium [Dulbecco’s Modified Eagle Medium (DMEM, GIBCO) with 10% FCS, penicillin (100 U/ml) and streptomycin (100 μg/ml)]. The next day, the medium was replaced and cells were cultured at 37 °C until confluent, when the cells were harvested and frozen.

### Production of recombinant GDF9 and BMP15

The HIV-1-based self-inactivating-type lentiviral vector plasmid pLV-EGFP was constructed by replacing the EGFP cDNA with mouse GDF9 or BMP15 cDNA, respectively, and isolated for infection of HEK293T cells^63^.

### *In vitro* follicle culture

Ovaries were collected from 12 day-old mice from which follicles were mechanically isolated prior to *in vitro* culture^64^. Each follicle was placed in a 30 μl droplet of DMEM (GIBCO) with 10% FCS, penicillin (100 U/ml) and streptomycin (100 μg/ml). The droplets were placed in 60 mm uncoated culture dishes (Iwaki) and covered with paraffin oil (Nacalai). After overnight culture, half of the culture medium was replaced by HEK293T cell-conditioned medium containing recombinant GDF9 or BMP15. Culture medium from non-transfected HEK293T cells was used as a negative control. Half of the medium was replaced every other day. Follicle volumes were calculated as described^65^.

### Purification of His tagged GDF9 or BMP15 secreted into MEF cell media

Mouse *Gdf9* or *Bmp15* cDNA tagged with Flag/Hisx6 at the C-terminus was inserted into a pNCAG vector consisting of the CAG-promoter and rabbit β globin poly-adenylation signal, respectively^56^. *Calr*^+/+^ or *Calr*^−/−^ MEF cells were cultured in 6-well plates and transfected with pNCAG-Gdf9 Flag/His or pNCAG-Bmp15 Flag/His using lipofectamine LTX (Invitrogen). After 18 hours, cell debris was removed by two washes in PBS (GIBCO) and the medium was replaced [DMEM with 3% FCS, penicillin (100 U/ml) and streptomycin (100 μg/ml)]. The culture medium was collected every 24 hours for 4 days. Purification of His tagged recombinant protein in the culture medium was performed using μMACS anti-His Isolation Kit (Miltenyi Biotec). Two milliliters of culture medium were used for purification and the proteins were eluted into 70 μl.

### Statistical analysis

The values were the means ± standard deviation or standard error of the mean from at least three independent experiments. Statistical analyses were performed using Student’s t-test. Differences were considered significant at P < 0.05.

## Additional Information

**How to cite this article**: Tokuhiro, K. *et al.* Calreticulin is required for development of the cumulus oocyte complex and female fertility. *Sci. Rep.* **5**, 14254; doi: 10.1038/srep14254 (2015).

## Supplementary Material

Supplementary Information

## Figures and Tables

**Table 1:** Fertility of *Calr* fcKO female mice after PMSG/hCG treatment.

Genotype	N^a^	Females^b^	Total eggs^c^	Normal eggs^d^	Fertilization rate^e^ (%)
*Calr*^fl/+^; *Zp3-cre*	4	5	28.2 ± 4.1	22.8 ± 6.8	70.2 ± 19.7
*Calr*^fl/−^; *Zp3-cre*	4	6	0.8 ± 1.0	0.5 ± 0.8	0

Data are presented as mean ± SD.

^a^number of replicates.

^b^number of mice used.

^c^number of ovulated eggs/animal.

^d^number of MII eggs/animal.

^e^number of 2 cell embryos/number of inseminated eggs.

**Table 2:** Ovarian characteristics after PMSG/hCG hormonal treatment.

Genotype	N^a^	Ovarian weight (mg)	Antral follicles^b^	Antral follicles with expanded cumulus^c^
*Calr*^fl/+^; *Zp3-cre*	6	7.88 ± 1.26	27.8 ± 5.7	26.2 ± 4.8
*Calr*^fl/-^; *Zp3-cre*	6	4.18 ± 1.36**	44.7 ± 11.5**	1.2 ± 1.5***

**P < 0.01, ***P < 0.001. Data are presented as mean ± SD.

^a^number of mice used.

^b^number of antral follicles/section.

^c^number of antral follicles with expanded cumulus/section.

**Table 3:** Development of *in vitro* matured and fertilized *Calr* fcKO oocytes.

Genotype	N^a^	Females^b^	MII/GV^c^ (% ± SD)	2cell/MII^d^ (% ± SD)	Pups/transferred^e^
*Calr*^fl/+^; *Zp3-cre*	7	11	320/585 (54.7 ± 21.0)	139/248 (55.6 ± 12.2)	ND
*Calr*^fl/−^; *Zp3-cre*	8	16	575/961 (63.7 ± 12.9)	147/471 (31.4 ± 14.6)	8/64# (12.5%)

Data are presented as mean ± SD.

^a^number of replicates.

^b^number of mice used.

^c^number of *in vitro* matured MII eggs/number of GV oocytes.

^d^number of 2 cell embryos/number of inseminated eggs.

^e^number of pups/number of transferred embryos.

^#^Eight pups were obtained from two foster mothers.
