# Supplementary material for: Calreticulin is required for development of the cumulus oocyte complex and female fertility
Source: Sci Rep. 2015 Sep 21;5:14254. doi: 10.1038/srep14254 (PMC4585710; doi:10.1038/srep14254)
Supplement: Supplementary Information [file srep14254-s1.doc]

**Supplementary Information**

**Calreticulin is required for development of the cumulus oocyte complex and female fertility**

Keizo Tokuhiro^1^, Yuhkoh Satouh^1^, Kaori Nozawa^1,2^, Ayako Isotani^1,3,4^, Yoshitaka Fujihara^1^, Yumiko Hirashima^5^, Hiroyuki Matsumura^1,4^, Kazuhiro Takumi^1,4^, Takashi Miyano^5^, Masaru Okabe^1^, Adam M. Benham^1,6^, and Masahito Ikawa^1,2,3,4^

^1^ Research Institute for Microbial Diseases, Osaka University, Suita, Osaka, Japan

^2^ Graduate School of medicine, Osaka University, Suita, Osaka, Japan

^3^ Immunology Frontier Research Center, Osaka University, Suita, Osaka, Japan

^4^ Graduate School of Pharmaceutical Sciences, Osaka University, Suita, Osaka, Japan

^5^ Graduate School of Agricultural Science, Kobe University, Kobe, Hyogo, Japan

^6^ School of Biological and Biomedical Sciences, Durham University, Durham, United Kingdom

**
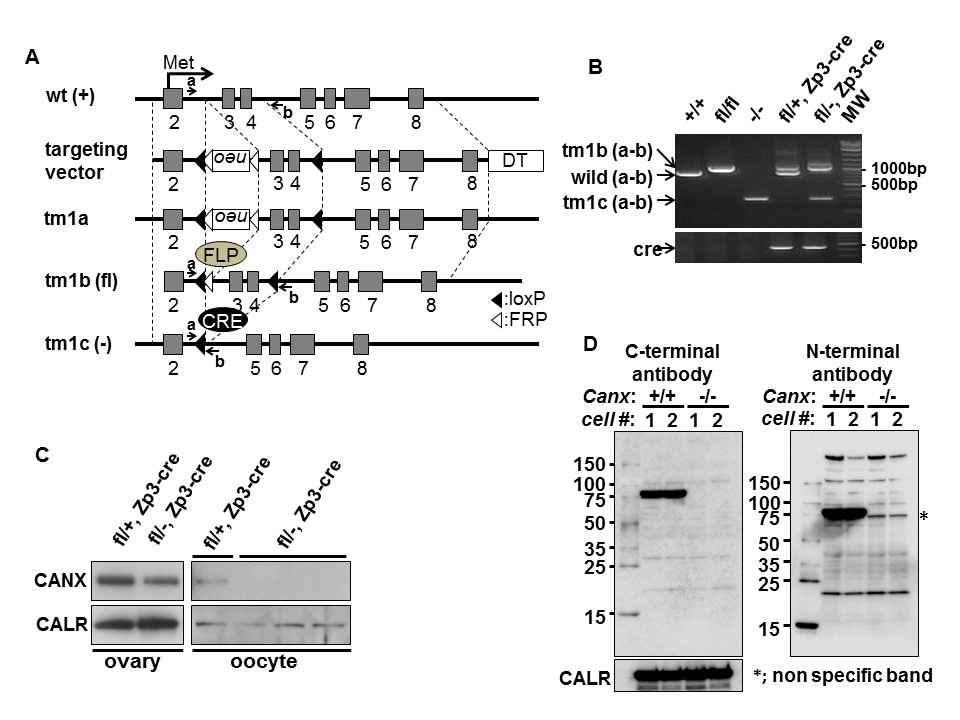
Figure S1. Generation of *Canx* KO and cKO mice**

**A**, Diagram of targeting strategy. The homologous recombinant, tm1a, contains two loxP sites (black arrowheads) flanking exons 3 and 4 as well as a neomycin-resistant cassette flanked by FLP recombinase recognition sites (white arrowheads). Mice heterozygous for the tm1a were mated with transgenic mice expressing FLP recombinase to remove the neo cassette (tm1b). Mating of tm1b mice with transgenic mice expressing the Cre recombinase resulted in the deletion of exon 3-4 and generated the KO allele (tm1c). **B**, The breeding strategy of cKO and KO was described in SI Materials and Methods. Offspring were genotyped by PCR with primers indicated in A (pr-a; 5’- GCTCTGGATGCCTTGGGATTTGATTTCC -3’ and pr-b; 5’- GGATTTAGTGGATATCCCCAGTATGAGC -3’). The amplicons corresponding to +, fl, and – are 851, 1062, and 250 bps, respectively. The Cre transgene was detected by PCR as a 450 bps band with primers, pr-CreF; 5’- gtttcactggttatgcggcgg -3’ and pr-CreR 5’- ttccagggcgcgagttgatag -3’. **C**, Constitutive expression of CALR was observed in ovary and oocyte lysates from *Canx* fcKO mice (*Canx* ^fl/-^; *Zp3-cre*) by immunoblot. CANX was oocyte-specific deficient in *Canx* cKO mice. **D**, The signal of truncated CANX was not detected in *Canx* deficient MEF cells. The asterisk indicates a non-specific band.


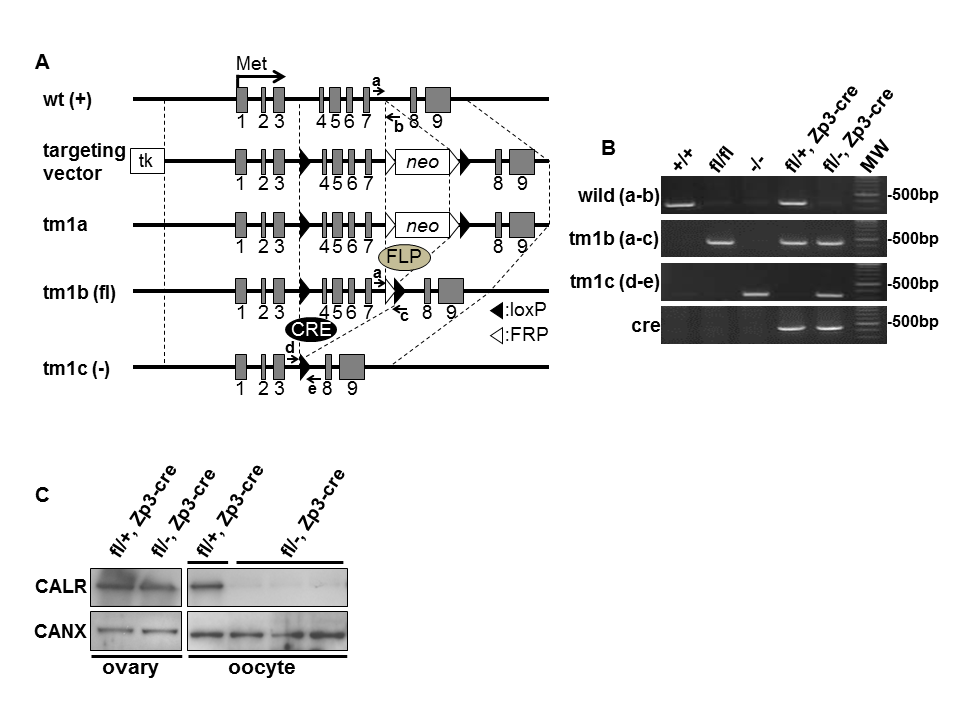


**Figure S2. Generation of *Calr* cKO mice**

**A**, Diagram of targeting strategy. The homologous recombinant, tm1a, contains two loxP sites (black arrowheads) flanking exons 4-7 as well as a neomycin-resistant cassette flanked by FLP recombinase recognition sites (white arrowheads). Mice heterozygous for the tm1a were mated with transgenic mice expressing FLP recombinase to remove the neo cassette (tm1b). Mating of tm1b mice with transgenic mice expressing the Cre recombinase resulted in the deletion of exons 4-7 and generated the KO allele (tm1c).　**B**, Offspring were genotyped by PCR with primers indicated in A (pr-a; 5’- gagtggaaaccacgtcaaattgacaacc -3’, pr-b; 5’- cttctctgataagttttcctctgacctc -3’, pr-c; 5’- agggttccggatccgatgaagttcc -3’, pr-d; 5’- ccagaatgctgatcttcataccatgg -3’, and pr-e; 5’- aatgaccagagttgattccaagg -3’). The amplicons corresponding to +, fl, and – are 373, 335, and 442 bps, respectively. **C**, CALR was oocyte-specific deficient in *Calr* cKO mice. Constitutive expression of CANX was observed in ovary and oocyte lysates from *Calr* fcKO mice (*Calr* ^fl/-^; *Zp3-cre*) by immunoblot.

**
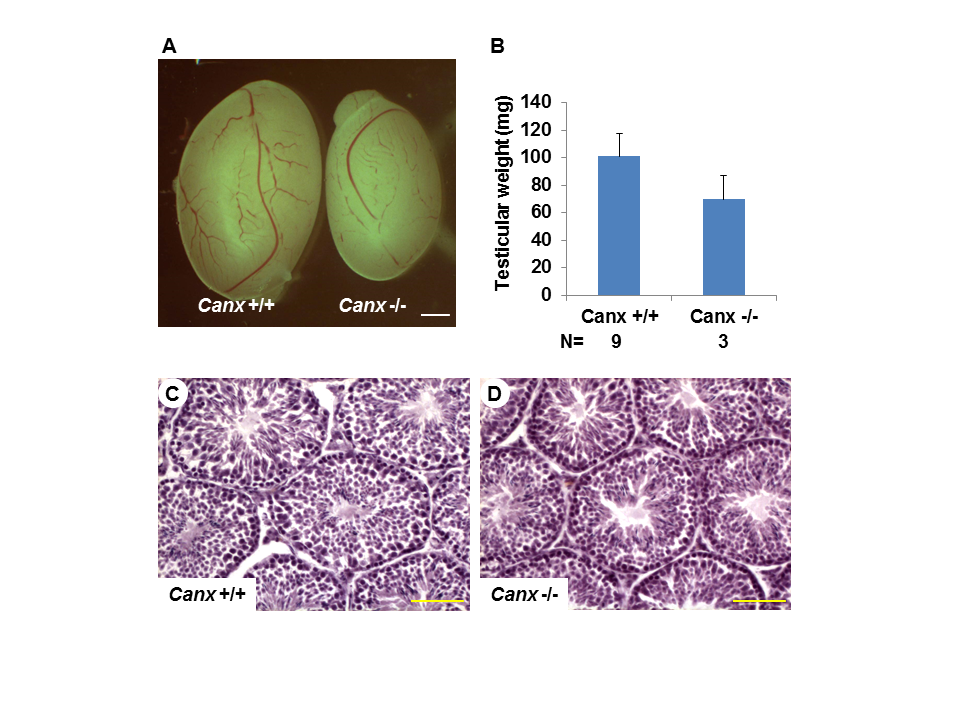
Figure S3. Spermatogenesis in *Canx* KO mice**

**A**, Testes from control and *Canx* KO male mice at 3 months of age. **B**, Testicular weight of control and *Canx* KO mice (101 ± 10 mg in +/+ and 70 ± 14 mg in -/-). Testicular weight in the *Canx* KO is reduced. **C and D**, Testicular sections (5µm) were prepared after 4% paraformaldehyde /PBS fixation and stained with hematoxyin and eosin. Spermatogenesis appears normal in the *Canx* KO. Scale bar=1mm (A) and 100µm (C and D).

**
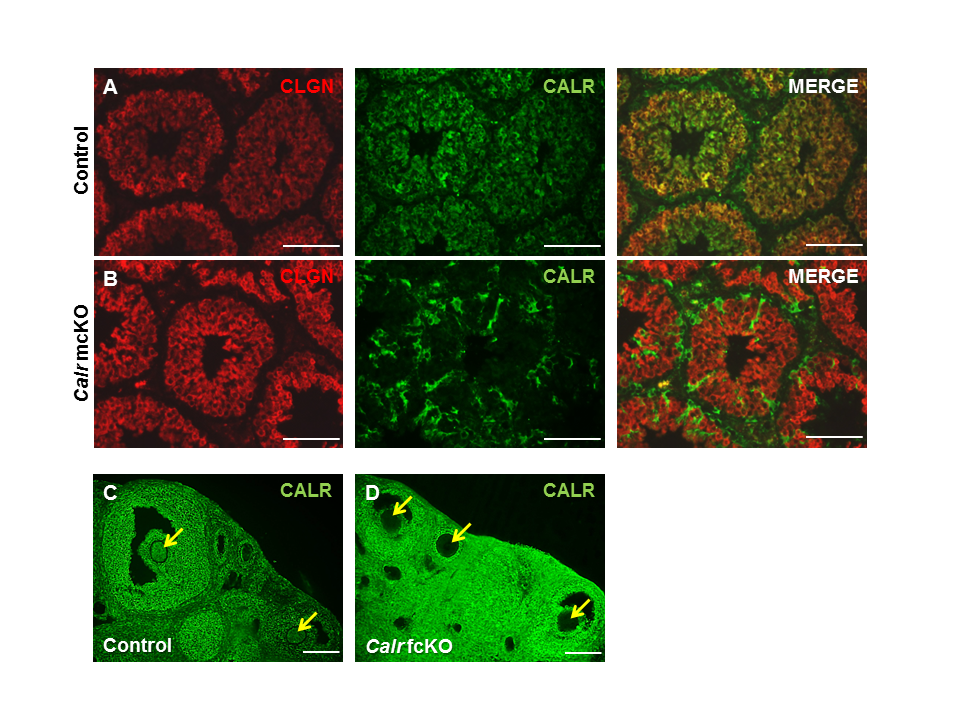
Figure S4. Germ cell-specific deletion of CALR from *Calr* mcKO**

**A and B**, Immunostaining of testicular sections from control (*Calr* ^fl/+^; *Stra8-cre*) and *Calr* mcKO (*Calr* ^fl/-^; *Stra8-cre*) mice with anti-CLGN and anti-CALR antibodies. CLGN was normally expressed in control spermatocytes and spermatids, but CALR was absent in almost all testicular germ cells in *Calr* mcKO mice, although present in somatic cells. **C and D**, CALR was ubiquitously expressed in ovarian cells from control animals (*Calr* ^fl/+^; *Zp3-cre*). In *Calr* fcKO (*Calr* ^fl/-^; *Zp3-cre*) mice, oocyte-specific absence of CALR was confirmed. Developing oocytes are indicated with arrows. Scale bar=100µm.

**
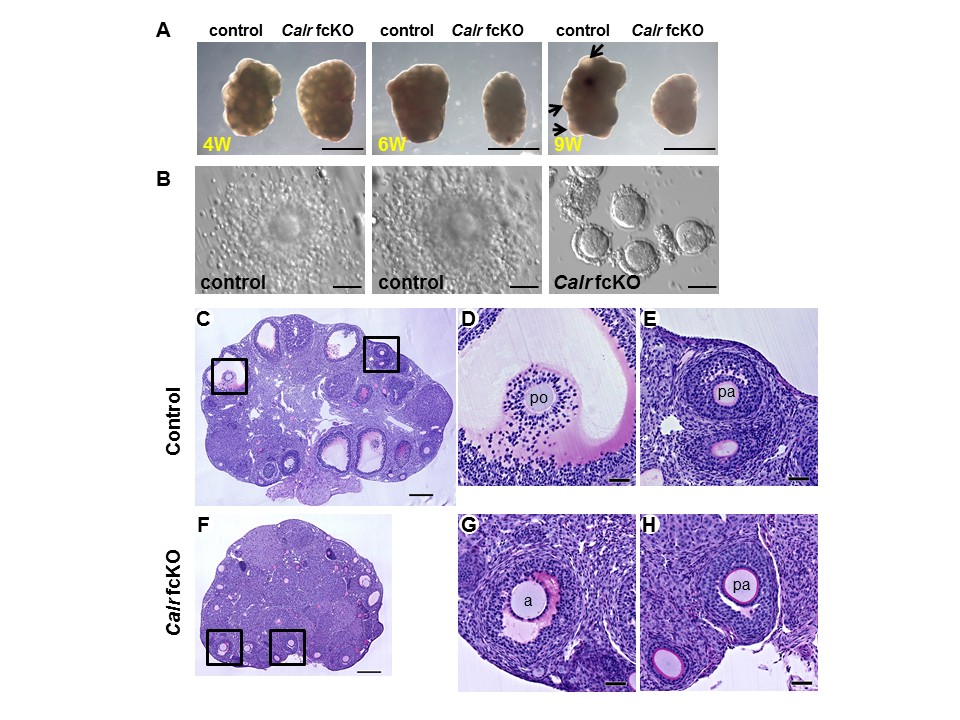
Figure S5. Abnormal folliculogenesis in *Calr* fcKO mice**

**A**, Gross morphology of control and *Calr* fcKO ovaries from 4 week-, 6 week- or 9 week-old mice at 48 hours after PMSG injection. In controls (*Calr* ^fl/+^; *Zp3-cre*), fully developed antral follicles were observed in 9 week-old ovaries (arrows), but in *Calr* fcKO (*Calr* ^fl/-^; *Zp3-cre*) the ovary surface was smooth and follicles were abnormal. Scale bar, 2 mm. **B**, Ten hours after HCG injection, COCs were collected from ovaries using 26G needles. The control oocytes were surrounded by fully grown and expanded cumulus cells layers. In the *Calr* fcKO mice, normal looking oocytes were recovered with a few packed cumulus cells. Scale bar, 50 µm. **C-H**, PAS/hematoxylin-stained ovarian sections of 12 week-old control (C-E) and *Calr* fcKO (F-H) mice. Antral (G) and preantral (E and H) follicles were found in control and *Calr* fcKO, preovulatory follicles (D) were found in control, not in *Calr* fcKO (D). po, preovulatory follicle; pa, preantral follicle; a, antral follicle; Scale bar, 300 µm (C and F) and 50 µm (D, E, G, and H).
